# Lifestyle instability: an overlooked cause of population obesity?

**DOI:** 10.1038/s41366-025-01787-5

**Published:** 2025-04-09

**Authors:** Arthur B. Daw, Chiara N. Hinchcliffe, Lewis J. James, James A. King

**Affiliations:** 1 Surpic Ltd, Cheltenham, UK; 2 National Centre for Sport and Exercise Medicine, School of Sport, Exercise and Health Sciences, Loughborough University, Loughborough, UK; 3 NIHR Leicester Biomedical Research Centre, University Hospitals of Leicester NHS Trust and University of Leicester, Leicester, UK

**Keywords:** Risk factors, Lifestyle modification

Obesity remains a pressing health challenge globally. While population prevalence may be stabilising in adults within some (high-income) nations [1], levels remain persistently high and are rising in children. Moreover, the proportion of individuals living with severe obesity is increasing [1]. Although advances in pharmacotherapy are revolutionising obesity management for those fortunate to have access, less progress has been made in addressing excess body fat gain as a public health issue. This is disappointing because individuals and society stand to gain more, in health and economic terms, from primary prevention of obesity. Whilst recognising powerful socio-cultural, political and economic influences, the lack of progress may also stem from the failure to tackle the root causes of excess body fat gain. Specifically, public discourse surrounding obesity development often assumes that individuals accumulate excess body fat over time in a subtle yet continuous (day-to-day; week-to-week) manner; implying (and necessitating) an enduring state of positive energy balance. However, accumulating research suggests this may not be the case [2]. Instead, excess body fat gain often results from large, relatively short-term episodes of positive energy balance (interspersed with longer periods of energy deficit and/or energy balance). These brief periods of energy imbalance and fat gain are triggered by ‘*disruptions’* to individuals day-to-day lives, resulting from a temporary but significant mis-match in energy intake and energy expenditure. Therefore, ‘*lifestyle instability’* may be an underappreciated risk factor for excessive body fat gain which has fundamental implications for obesity prevention strategies and public health.

The idea that excess body fat gain progresses continuously, albeit subtly, stems from observational studies documenting population weight change over time (years). With infrequent body mass measurements (e.g., annually), basic calculations provide estimates of average body fat gain in populations over extended periods [3]. These data can be extrapolated to estimate daily energy imbalance, necessary to explain annual body fat change. These calculations are often read to imply that body fat gain has occurred due to energy intake exceeding expenditure by a dozen or so kilocalories each day (equivalent to eating a few grapes) [3]. In a public health context, this narrative emphasises the need for individuals to address relatively small day-to-day eating and activity behaviours underpinning excess body fat accumulation over time [4].

A problem with this concept is that it is based on data lacking granularity (i.e., infrequent body mass/composition measurements). Recent technology developments now make it possible to generate rich datasets including measurements of body mass and composition on a daily basis [2]. Such data has uncovered significant variability in body mass, shown to deviate within weeks (mostly representing changes in body water) and between seasons [2]. Importantly, large increases in body mass can be seen following lifestyle disruptions, including holidays and celebratory/festive periods [5], likely driven by altered eating behaviours. Notably, the magnitude of body fat gain provoked by these events may fully account for annual body fat gain. Therefore, it is possible that body fat gain proceeds in an intermittent, stepwise fashion, when compensatory responses (biological and behavioural) do not fully reverse fat gain provoked by episodic lifestyle disruptions. Globally, individuals are exposed to such events regularly, with successive occasions having the potential to compound body fat accumulation. In the UK, a leading example of a disruptor is the Christmas period where daily energy intake can increase to levels reported by Tour de France cyclists (6000 kcal/day) [6, 7] (despite unchanged or even decreased movement related energy expenditure).

Beyond these recurring lifestyle disruptions, are less frequent, yet potentially more destabilising life events, with potential to exacerbate body fat gain. These include changes to living arrangements, relationships, careers, health status (including medication courses), injuries and parenthood [8]. Each of these examples has the potential to temporarily modulate energy balance by influencing eating and activity behaviours. One well studied example of this phenomenon is the body mass gain (appreciating the majority of mass change will be fat) observed when young adults start university, often referred to as the ‘*freshman 15’* but empirically determined as several pounds (3–4 kg) [9].

The primary implication of this thesis is that greater focus on disruptive lifestyle events and lifestyle instability may help individuals avoid excess body fat gain with ageing. Tangibly, this requires individuals having a better awareness and understanding about when they are at high-risk of gaining excess body fat. People also need more information to support their dietary and movement-related decisions affecting energy balance. At a population level, the most cost-effective way for this to happen is likely via new technologies that facilitate self-management. For example, commercial platforms (apps) already permit the monitoring of day-to-day energy balance through the integration of energy intake (diet tracking), energy expenditure (device-measured movement related energy expenditure integrated with estimates of resting metabolic rate and diet-induced thermogenesis), body mass and body composition data (i.e., ‘*smart scales’*) [10]. Going forward, machine learning will improve these systems through behavioural pattern recognition. Moreover, artificial intelligence will enhance personalisation of support via one-to-one (interactive) coaching. The utility of these systems will rest upon open questions about user interest, engagement and access.

It is hoped the ideas in this article will prompt further research. This includes the development of large datasets containing information about daily body composition, and dietary/movement behaviours over time. This will facilitate a more accurate understanding of energy balance dynamics and fat variation around key life events. These data will help determine the relative importance of disruptors and lifestyle instability as a cause of long-term body fat gain. To achieve this, research must document the magnitude and consistency of fat variation around events; and importantly, the extent to which body fat gain is reversed after disruptive events. Creating large and diverse datasets will help identify groups most vulnerable to excess fat gain from lifestyle disruptions, whether due to demographic factors, genetic predisposition, metabolic differences, or behavioural traits.

This article has two key implications for public health. First, if lifestyle disruptors are the main driver of annual fat gain, prevention strategies should focus on these events. Second, if fat gain occurs in short episodes, effective interventions may only require infrequent temporary behavioural changes.
